# Early Hypertriglyceridemia and Respiratory Outcomes in Preterm Neonates

**DOI:** 10.3390/nu18193163

**Published:** 2026-09-25

**Authors:** Maria Di Chiara, Flavia Gloria, Ludovica Toro, Eleonora Cresta, Gianluigi Laccetta, Francesca Lucci, Raffaella Cellitti, Gaia Loffredo, Gianluca Terrin

**Affiliations:** Department of Maternal and Child Health, Policlinico Umberto I, Sapienza University, 00161 Rome, Italyeleonora.cresta@uniroma1.it (E.C.); gianluigi.laccetta@uniroma1.it (G.L.); gianluca.terrin@uniroma1.it (G.T.)

**Keywords:** Hypertriglyceridemia, preterm neonates, bronchopulmonary dysplasia, enteral feeding, parenteral nutrition, neonatal morbidity

## Abstract

**Background:** Hypertriglyceridemia (HiTG) is a frequent metabolic complication in preterm neonates receiving parenteral nutrition (PN), with potential implications for respiratory outcomes. However, predisposing factors and the clinical impact of HiTG in this population remain poorly defined. **Objectives:** To identify factors predisposing preterm neonates to HiTG development and to assess the association between HiTG and neonatal respiratory morbidity. **Methods:** A retrospective matched cohort study was conducted, nested within a single-center cohort of neonates receiving routine parenteral nutrition (PN) and on respiratory support, with gestational age ≤ 32 weeks and/or a birth weight ≤ 1500 g, consecutively admitted between January 2015 and May 2023 into the Neonatal Intensive Care Unit (NICU) of ‘Umberto I’ University Hospital, Sapienza University of Rome. HiTG was defined as at least one serum triglyceride (TG) value > 180 mg/dL during the first 7 days of life. TG levels were measured from morning samples collected alongside routine laboratory draws; for borderline values (180–200 mg/dL), a confirmatory sample was obtained after a 4 h lipid-free interval. Neonates with HiTG were classified as cases; newborns without HiTG served as controls, matched 1:1 for gestational age and birth weight. **Results:** A total of 110 neonates were enrolled (55 cases, 55 controls). HiTG was independently associated with bronchopulmonary dysplasia (BPD) (OR 29.46; 95% CI 5.82–149.07; *p* < 0.001) and prolonged invasive mechanical ventilation (pIMV) (OR 4.85; 95% CI 1.69–13.89; *p* = 0.003), after adjustment for surfactant administration and extremely low birth weight. Time to achieve full enteral feeding (FEF) was independently associated with HiTG development (β = +0.153; *p* < 0.001), whereas parenteral lipid intake during the first week of life was inversely associated (β = −0.081; *p* = 0.039). **Conclusions:** HiTG is independently associated with BPD and prolonged invasive mechanical ventilation in preterm neonates. Parenteral lipid intake during the first week was not higher in infants who developed HiTG. Delayed achievement of FEF was also associated with HiTG, but it occurred after the exposure window and cannot therefore be regarded as a predisposing factor. These findings are hypothesis-generating. Randomized trials are needed to assess whether closer monitoring of triglyceride levels and early advancement of enteral feeding in preterm neonates may help reduce BPD.

## 1. Introduction

Preterm neonates on routine parenteral nutrition (PN) are at high risk of developing hypertriglyceridemia (HiTG), particularly during the first week of life, with reported incidence rates ranging from 25% to 50% depending on gestational age and lipid dose [1,2]. HiTG may impair pulmonary gas exchange and has been associated with adverse respiratory and metabolic outcomes in this vulnerable population [1,3,4]. Recent studies have further characterized the risk factors and impact of HiTG in preterm infants under 32 weeks [5,6].

PN is essential to meet the nutritional requirements of preterm neonates during the early postnatal period [7,8]. Intravenous lipid emulsions are a key component of PN, providing up to 50% of the non-protein energy intake [9,10].

However, the administration of lipid emulsions carries the risk of HiTG, which may in turn contribute to adverse clinical outcomes.

Sinclair et al. reported a high prevalence of hypertriglyceridemia in extremely preterm infants receiving parenteral lipid emulsions [11]. This aspect is still controversial in newborns. In adult patients, HiTG has been associated with impaired respiratory function, including decreased oxygenation and increased pulmonary vascular resistance [12,13]. In the neonatal setting, preliminary evidence suggests that lipid microemboli may deposit in the pulmonary vasculature, activate inflammatory mediators, and increase pulmonary vascular resistance, potentially contributing to lung injury [4,14]. However, the relationship between HiTG and respiratory outcomes in preterm neonates remains insufficiently investigated.

There are conflicting data about predisposing risk factors for HiTG in preterm neonates undergoing PN [1,4]. The association between HiTG and respiratory outcomes, such as bronchopulmonary dysplasia (BPD), has only been explored in small studies, resulting in limited and inconsistent data [1,3,15,16]. A comparison of the published studies that have investigated this association in very preterm or very low birth weight infants, including the population studied and the maximum intravenous lipid dose administered in each of them, is provided in Table S1.

In light of these considerations, we designed a retrospective matched cohort study, nested within the cohort of preterm neonates consecutively admitted to our unit [17,18], with two aims: (1) to identify predisposing factors for HiTG development in preterm neonates; and (2) to assess the association between HiTG and neonatal respiratory morbidity, including bronchopulmonary dysplasia (BPD) and prolonged invasive mechanical ventilation (pIMV).

## 2. Materials and Methods

### 2.1. Study Design and Population

This retrospective matched cohort study was nested within the single-center cohort of preterm neonates consecutively admitted to the Neonatal Intensive Care Unit (NICU) of ‘Umberto I’ University Hospital, Sapienza University of Rome, between January 2015 and May 2023, previously described by our group [19]. Part of the cohort analyzed here has been included in a previous case–control study from our group [1], which enrolled neonates from January 2017 to November 2019 and defined hypertriglyceridemia as a serum triglyceride value above 150 mg/dL. The present analysis differs in design, threshold and objectives. Eligibility was restricted to preterm neonates with a gestational age (GA) ≤ 32 weeks and/or a birth weight (BW) ≤ 1500 g who were receiving routine parenteral nutrition (PN) and required respiratory support during the first week of life. Neonates were ineligible if any of the following applied: presence of major congenital malformations, inborn errors of metabolism, congenital or acquired immunodeficiency, or congenital infections [20]; incomplete clinical records; or early outcome events, death or transfer to another hospital, occurring within the first 72 h of life.

Hypertriglyceridemia (HiTG) was defined as at least one serum triglyceride (TG) value > 180 mg/dL during the first 7 days of life. This cut-off was selected based on our research group’s prior institutional experience in preterm infants receiving intravenous lipid emulsions and on preliminary clinical observations suggesting clinically relevant elevations around this range [5,10]. TG thresholds around 180 mg/dL have been used in neonatal studies evaluating lipid tolerance and respiratory outcomes in preterm infants on PN [21]. According to our internal NICU protocol, serum TG monitoring is routinely initiated from the third day of PN and then repeated at regular intervals (typically every 72 h) during the first week of life. Blood samples were collected in the morning, simultaneously with routine laboratory draws, to minimize procedural stress and blood loss. For TG values in the borderline range (180–200 mg/dL), a confirmatory sample was obtained after a 4 h lipid-free interval, at the time of the next lipid bag change, to confirm the elevation and guide clinical decision-making, consistent with current ASPEN recommendations [22].

According to HiTG occurrence, we defined cases as neonates with the diagnosis of HiTG, whereas controls were neonates who did not develop HiTG.

Individual 1:1 matching was performed: for each case, one control was selected from the pool of eligible neonates without HiTG, matched for GA (within ±6 days) and BW (within ±100 g). No formal a priori sample size calculation was performed. The sample size was determined by the number of consecutive eligible neonates who developed HiTG during the study period. The exposure of interest was HiTG during the first 7 days of life, and the co-primary outcomes were BPD and prolonged invasive mechanical ventilation, both ascertained after the exposure window.

Parenteral nutrition was initiated within the first 24 h after NICU admission and delivered through a central venous catheter in all infants, ensuring adequate intake of fluids, electrolytes, and macronutrients until full enteral feeding (FEF, defined as 120 kcal/kg/day) was achieved [19]. A single lipid emulsion was used throughout the study period (Intralipid 20%, Fresenius Kabi, Bad Homburg, Germany), with no changes in formulation or manufacturer across enrolled neonates. Intravenous lipid emulsion was started at 1 g/kg/day and increased by 0.5 to 1 g/kg/day according to tolerance, up to a maximum of 3.5 g/kg/day by the seventh day of life. Lipids were administered by continuous infusion over 24 h, corresponding to a maximum infusion rate of approximately 0.15 g/kg/hour.

### 2.2. Data Collection

Clinical data were collected prospectively from birth to hospital discharge and subsequently reviewed retrospectively, covering both prenatal and postnatal characteristics as well as anthropometric parameters (raw measurements and standardized values). Crown-to-heel length was assessed at birth using a neonatal stadiometer, and occipitofrontal head circumference was measured with a non-elastic measuring tape. Weight, length, and head circumference were expressed as sex-specific percentiles and Z-scores derived from the Italian Neonatal Study (INeS) growth charts [23]. Extrauterine growth restriction (EUGR) was defined as a decrease of more than 1.0 in weight Z-score between birth and 36 weeks of postmenstrual age, in line with prior work evaluating the effect of parenteral nutrition on growth trajectories in preterm neonates [24,25,26].

Additionally, data on the main neonatal morbidities related to prematurity, such as necrotizing enterocolitis (NEC) stage of Bell ≥ 2, intraventricular hemorrhage (IVH) ≥ 2, periventricular leukomalacia (PVL), late-onset sepsis, defined as clinical sepsis and/or positive blood culture occurring after 72 h of life, retinopathy of prematurity (ROP) ≥ 2, and bronchopulmonary dysplasia (BPD) were also retrieved [27,28,29,30,31,32,33,34]. BPD was defined as the need for oxygen supplementation at 36 weeks postmenstrual age (PMA) according to Jobe and Bancalari [35,36]. Prolonged invasive mechanical ventilation (pIMV) was defined as the need for invasive mechanical ventilation for ≥7 days of life, as previously reported [1].

### 2.3. Statistical Analysis

Statistical analysis was performed using the Statistical Package for the Social Sciences Software for Microsoft Windows (SPSS Inc., Chicago, IL, USA), version 27.0. We checked for normality using the Shapiro–Wilk test. Continuous variables with a normal distribution were summarized as mean with 95% confidence interval and compared using the Student’s *t*-test. Other continuous variables were summarized as median and interquartile range and compared using the Mann–Whitney U test. We used the chi-squared test for categorical variables, or Fisher’s exact test when any expected cell count was below 5. The level of significance for all statistical tests was 2-sided (*p* < 0.05).

Associations between candidate covariates and HiTG (dichotomous outcome) were examined using a multivariable linear probability model, with heteroscedasticity-consistent (robust) standard errors to account for the non-constant variance intrinsic to binary outcomes. Binary logistic regression models were used for the subsequent outcomes of bronchopulmonary dysplasia (BPD) and prolonged invasive mechanical ventilation (pIMV), given the substantially different baseline prevalence of these outcomes (<50%). Results are reported as standardized β coefficients with 95% confidence intervals for HiTG, and as odds ratios with 95% CI for BPD and pIMV.

Candidate covariates were selected based on clinical relevance, those that are well-established in the literature as being associated with the dependent variable, and/or those that were statistically significant in preliminary univariate analyses. Candidate covariates were subjected to a stepwise selection procedure; backward elimination and forward selection yielded identical models, and hypertriglyceridemia, the exposure of interest, was retained in all models. The number of covariates in each final model was limited in accordance with the criterion of approximately one variable per ten events. The assumption of a linear relationship between the continuous covariates and the outcome was examined by adding a quadratic term and by fitting a restricted cubic spline with three knots placed at the 10th, 50th and 90th percentiles. Multicollinearity was assessed (e.g., tolerance and variance inflation factors), and highly correlated variables were not simultaneously included in the same model. Results are reported as odds ratios (OR) with 95% confidence intervals (CI) and two-sided *p*-values. Prolonged invasive mechanical ventilation was not entered as a covariate in the model for BPD, because it lies on the causal pathway between early respiratory morbidity and BPD; a sensitivity analysis including it is reported in the Results.

The statistician was blinded to the study aims. Neonates with incomplete clinical data were excluded a priori (see exclusion criteria); therefore, a complete-case analysis was performed on the remaining study population.

## 3. Results

We enrolled 110 neonates in the study: 55 cases compared to 55 controls. Of the 462 preterm neonates of the source cohort assessed for eligibility, 416 were eligible after application of the exclusion criteria; 55 of them developed HiTG during the first 7 days of life and were included as cases, and 55 controls were individually matched to them from the 361 eligible neonates who did not develop HiTG. The flow of the study population is shown in Figure 1.

Baseline prenatal and postnatal characteristics are presented in Table 1 and Table 2.

No statistically significant differences were found between cases and controls, except for the need for transfusion (69.1 vs. 52.7; *p* < 0.05) (Table 2). Nutritional characteristics during the first week of life are presented in Table 3. Variables extending beyond the exposure window are reported here rather than in Table 3. Enteral nutrition was started at a mean of 5.0 days of life (95% CI 2.7 to 7.4) in cases and 3.0 days (95% CI 2.2 to 3.8) in controls (*p* = 0.109), corresponding to a mean postmenstrual age of 28.0 weeks (95% CI 27.3 to 28.6) and 28.4 weeks (95% CI 27.7 to 29.0), respectively (*p* = 0.438). Full enteral feeding (FEF) was achieved at a mean of 32.6 days (95% CI 26.8 to 38.3) in cases and 23.0 days (95% CI 20.1 to 25.9) in controls (*p* = 0.004).

Multivariable linear regression analysis showed that the time to achieve FEF was independently associated with HiTG (β = +0.153; 95% CI +0.082 to +0.225; *p* < 0.001). Parenteral lipid intake during the first week of life was also retained in the model, with an inverse association (β = −0.081; 95% CI −0.159 to −0.004; *p* = 0.039). Gestational age and the day of life at which enteral nutrition was started were evaluated as candidate covariates and were not retained.

The main morbidities observed during the neonatal clinical course are summarized in Table 4. Length of hospital stay did not differ significantly between groups. Because this variable was markedly right-skewed, it is summarised as median with interquartile range and compared with the Mann–Whitney U test: 85 days (IQR 63 to 107) in cases and 74 days (IQR 57 to 88) in controls (*p* = 0.065).

Overall morbidity was significantly higher in cases compared to controls (89.1% vs. 74.5%; *p* = 0.041; Table 4). Regarding the respiratory outcomes, the proportion of neonates who developed BPD was significantly higher in cases than in controls, and the proportion of neonates who underwent prolonged invasive mechanical ventilation (pIMV) was significantly higher in those with HiTG than in controls (Figure 2).

Binary logistic regression showed that HiTG was independently associated with BPD, alongside surfactant administration and ELBW status (Figure 3). In a sensitivity analysis including prolonged invasive mechanical ventilation as an additional covariate, prolonged ventilation was not associated with BPD (OR 2.50; 95% CI 0.69 to 9.05; *p* = 0.162) and the estimate for HiTG was essentially unchanged (OR 25.23; 95% CI 4.88 to 130.48; *p* < 0.001).

Similarly, HiTG was independently associated with prolonged invasive MV, alongside surfactant administration and ELBW status (Figure 4).

## 4. Discussion

Our study showed that hypertriglyceridemia (HiTG), diagnosed during the first week of life, is independently associated with adverse respiratory outcomes, including bronchopulmonary dysplasia (BPD) and prolonged invasive mechanical ventilation (pIMV), in a cohort of preterm neonates with BW ≤ 1500 g and/or GA ≤ 32 weeks receiving parenteral nutrition and respiratory support. Parenteral lipid intake during the first week of life was not higher in the infants who developed HiTG; if anything, it was slightly lower, plausibly reflecting a more cautious advancement of intravenous lipids in the most immature infants. Delayed achievement of full enteral feeding was also associated with HiTG; however, since full enteral feeding was attained well beyond the first week of life, this association cannot be interpreted as a predictive or causal relationship.

Regarding the primary endpoint, few studies in the neonatal population have explored the association between HiTG and respiratory outcomes. Boscarino et al., in a retrospective case–control study, demonstrated that HiTG, defined as any serum triglyceride value above 150 mg/dL measured during lipid infusion, was associated with an increased risk of prolonged invasive MV (≥7 days); however, a higher occurrence of BPD in the HiTG group was not confirmed by their multivariate analysis [1]. Chan B. et al. reported that neonates with triglyceride blood levels of 180–400 mg/dL had an increased risk of BPD and more prolonged invasive MV compared to those with levels < 180 mg/dL [21]; however, our study demonstrated significant associations even at lower triglyceride thresholds. We also confirmed that ELBW status and surfactant administration are established risk factors for both BPD and prolonged invasive MV, consistent with the existing literature [39,40].

It has been widely reported that the amount of lipid administration is an established factor associated with the development of HiTG [4,6,14].

Giretti et al. in a retrospective study reported that IV lipid intake is independently associated with HiTG in preterm neonates [14]. Choi et al. in a retrospective study found that lipid intolerance was significantly associated with IV lipid dose [41].

Furthermore, our results do not support a positive association between the amount of PN lipids received within the first week of life and the occurrence of HiTG. Chan B et al. performed a simple comparison between neonates with different TG blood levels receiving lipids through PN and found that the amount of lipids received did not influence the risk of HiTG [21]. Our multivariable analysis is consistent with that observation: lipid intake was not higher in the infants who developed HiTG, and was in fact slightly lower, which we interpret as reflecting the more cautious advancement of lipids in the most immature infants rather than a protective effect of lower intake.

Also, Choi et al. evaluated intravenous lipid administration within the first 28 days of life and found that neonates with HiTG received significantly higher doses of lipids through PN; however, we focused our study on the first week of life, which is a critical window for preterm neonates, and this result showed a more precise understanding of the role of early lipid administration in HiTG development [41].

In a prospective observational study of 75 ELBW infants on a standardized parenteral lipid escalation protocol, Holtrop et al. reported that elevated triglyceride levels were associated with lower birth weight rather than with lipid dose [42]. However, triglyceride levels were measured after certain IV lipid doses were reached, and the exact timing of the sampling is unclear, while in our study, we performed blood sampling during the first week of life.

The association between HiTG and delayed achievement of full enteral feeding has not been specifically explored in previous studies. Since full enteral feeding was achieved at a mean of 32.6 days in cases and 23.0 days in controls, that is, well after the first week of life in which HiTG was ascertained, the temporal sequence does not allow delayed full enteral feeding to be considered a predisposing factor for HiTG. Most probably, both early HiTG and the delayed attainment of full enteral feeding reflect greater underlying immaturity and illness severity.

One possible interpretation of the association between HiTG and the later attainment of FEF is that early enteral nutrition may mitigate the metabolic complications associated with parenteral nutrition, including HiTG; this remains a hypothesis that the present design cannot test [43,44].

Our speculation is consistent with previous results. As demonstrated by our group in a prospective cohort study, the Early-Feeding cohort, who received MEF within the first 7 days of life, showed significantly lower rates of metabolic complications compared to the Late-Feeding cohort, who started MEF later in life [44]. The authors speculate that PN could be associated with the development of intestinal atrophy, reduced gastrointestinal blood flow, digestive dysfunction, and delayed development of the mucosal epithelium, and that these factors could impair tolerance to PN and contribute to metabolic complications. In this regard, short-chain fatty acids such as butyrate, produced through enteral feeding fermentation, have demonstrated beneficial effects on intestinal homeostasis and may contribute to mitigating the metabolic complications associated with prolonged PN [45].

In the univariate analysis, the HiTG group showed a significantly higher rate of blood transfusions compared to controls. Although this association was not assessed in a multivariate model, as transfusions were not a primary outcome of the study, it is biologically plausible. Previous studies have shown that higher levels of HiTG correlate with increased hemolysis [46,47]. Dimeski et al. suggested that elevated lipid concentrations might disrupt the lipid structure of the erythrocyte membrane, increasing membrane fragility, reducing circulating red blood cell mass, and consequently leading to a greater need for transfusions [47].

Moreover, some studies have demonstrated an increased risk of comorbidities associated with blood transfusions in preterm neonates [48,49].

Notably, the composite morbidity endpoint was significantly higher in neonates with HiTG compared to controls (Table 4), suggesting that HiTG may represent a marker of greater overall clinical vulnerability in preterm neonates, beyond respiratory outcomes alone.

Metabolic analysis of infants with BPD has shown distinct nutritional and metabolic profiles under early nutrition therapy [50,51,52].

As already described, particularly in studies conducted on adult populations, we hypothesized that the pathogenic mechanisms may be that HiTG might increase the risk of developing lipid micro-emboli, potentially contributing to respiratory distress and lung parenchyma damage [4,53]. Furthermore, Prasertsom et al. demonstrated that lipid emulsions increased pulmonary vascular resistance in preterm infants, probably due to variation in eicosanoid metabolism [54]. These factors may contribute to the need for prolonged MV in neonates with HiTG. Additionally, lipids in the intracapillary or alveolar space may activate inflammatory mediators, potentially promoting tissue inflammation that could be associated with lung damage and contribute to BPD development [12,55]. Moreover, oxidative stress has been recognized as a contributing pathway to organ damage in vulnerable neonatal populations, and HiTG may further amplify this oxidative burden [56]. However, these pathophysiological mechanisms remain hypothetical and require confirmation through dedicated experimental studies.

From a clinical perspective, our findings suggest that preterm neonates receiving parenteral nutrition should be closely monitored for hypertriglyceridemia, particularly during the first week of life. Random triglyceride measurements have been shown to be a valid monitoring tool in this clinical setting [57]. Early identification of HiTG may prompt timely adjustments in lipid infusion rates or facilitate an earlier transition to enteral nutrition [6]. The independent association between HiTG and adverse respiratory outcomes underscores the importance of integrated nutritional and respiratory management strategies in the NICU setting. Whether an earlier attainment of full enteral feeding influences the occurrence of HiTG cannot be established from our data and would need to be addressed prospectively [58]. Future multicenter prospective studies with larger sample sizes are warranted to confirm these associations and to evaluate whether targeted nutritional interventions can reduce the incidence of HiTG-related respiratory morbidity.

Our study presents several methodological strengths. First, the 1:1 caliper matching for gestational age (±6 days) and birth weight (±100 g) ensured adequate comparability between the two groups, minimizing confounding by these key prognostic variables. Second, all covariates included in the multivariate models were selected a priori based on a dual criterion of statistical significance at univariate analysis and clinical plausibility supported by the existing literature, and collinearity diagnostics (variance inflation factor) confirmed the independence of all predictors in each model. Third, data collection followed a predefined protocol; the classification of cases and controls was conducted by consensus among three researchers to reduce observer bias, and the statistical analysis was performed by a blinded statistician. Finally, by focusing on triglyceride measurements during the first week of life, this study provides a more precise characterization of early lipid metabolism in preterm neonates compared to previous studies that used later or variable sampling windows.

Our findings should be interpreted with consideration of several limitations.

Precision is limited by the sample size: only associations of substantial magnitude could be detected, and the confidence intervals are wide; matching for gestational age and birth weight controlled for the two dominant confounders, and the estimate for hypertriglyceridemia remained stable across the models tested. Cumulative parenteral lipid intake is in part determined by the outcome, since infusion was reduced once hypertriglyceridemia was detected; in the days preceding the first determination, intake did not differ between groups, so it cannot be read as a determinant. The retrospective single-center design limits external validity and leaves residual confounding possible. Immortal time bias does not arise, since both outcomes were ascertained after a fixed exposure window. The date of onset of sepsis and acute kidney injury were not recorded, and triglyceride monitoring beyond the first week followed clinical indication, so their concurrence with hypertriglyceridemia could not be assessed. Confirmation in a prospective multicenter study is required.

To limit observer bias, the data for the analysis were collected by researchers not involved in the eligibility assessment and who were unaware of the study outcome and design. We discussed and defined a protocol for the collection, measurement and interpretation of data before starting the study. Besides, a blinded statistician performed the data analysis. With only 55 cases and 55 controls, the study might lack statistical power to detect subtle differences, leading to wide confidence intervals and reducing the reliability of conclusions. The sample size was not based on a formal power calculation but was determined by the number of eligible cases identified during the study period. Thus, further larger prospective studies are warranted. Multicenter collaboration would be essential to achieve adequate statistical power and improve the external validity of the findings. Additionally, neonates who died or were transferred before 72 h of life were excluded, as serum TG monitoring in our NICU protocol is initiated from the third day of parenteral nutrition, making HiTG classification unfeasible before this time point. While this exclusion was methodologically necessary, it may have introduced a survival bias by excluding the most critically ill neonates. Furthermore, although our protocol standardized morning sampling alongside routine laboratory draws, TG measurements were obtained under non-fasting conditions, as a true fasting state is not achievable in preterm neonates receiving continuous PN. This inherent variability in TG levels should be considered when interpreting our findings. Finally, although each logistic regression model addressed a distinct a priori clinical hypothesis, no formal correction for multiple comparisons was applied; therefore, the possibility of type I error cannot be entirely excluded, and results should be confirmed in larger studies. Some potential sources of bias inherent in this design deserve mention. Neonates who died or were transferred within the first 72 h of life could not be classified for the exposure and were therefore ineligible, which limits the generalizability of our findings to the most severely ill infants; among the neonates analyzed, early death was infrequent and equally distributed between cases and controls. Standardized differences for the baseline variables reported in Table 1 and Table 2 showed a residual imbalance in the direction of slightly greater immaturity among cases, mainly for complete antenatal corticosteroid prophylaxis and gestational age; the former is adjusted for in both multivariable models and the latter was a matching variable. Finally, cumulative parenteral lipid intake and late-onset sepsis were not entered in the models for the respiratory outcomes, since both follow or are affected by the exposure, and conditioning on them would not estimate the total effect of hypertriglyceridemia; a sensitivity analysis including late-onset sepsis left the estimates essentially unchanged.

## 5. Conclusions

In this retrospective matched cohort study, HiTG diagnosed during the first week of life was independently associated with BPD and prolonged invasive mechanical ventilation in preterm neonates with BW ≤ 1500 g and/or GA ≤ 32 weeks. Early HiTG was also associated with a delayed achievement of full enteral feeding, an association not previously described in the neonatal literature; since full enteral feeding was attained after the ascertainment of HiTG, this finding is hypothesis-generating and does not identify a risk factor.

Given the retrospective design and the modest sample size, these associations require confirmation in larger prospective multicenter studies. Nonetheless, our findings support the clinical relevance of monitoring serum triglyceride levels during the first week of life in preterm neonates receiving parenteral nutrition, and generate the hypothesis, to be tested in prospective studies, that nutritional strategies aimed at reducing early HiTG may influence respiratory morbidity.

## Figures and Tables

**Figure 1:**
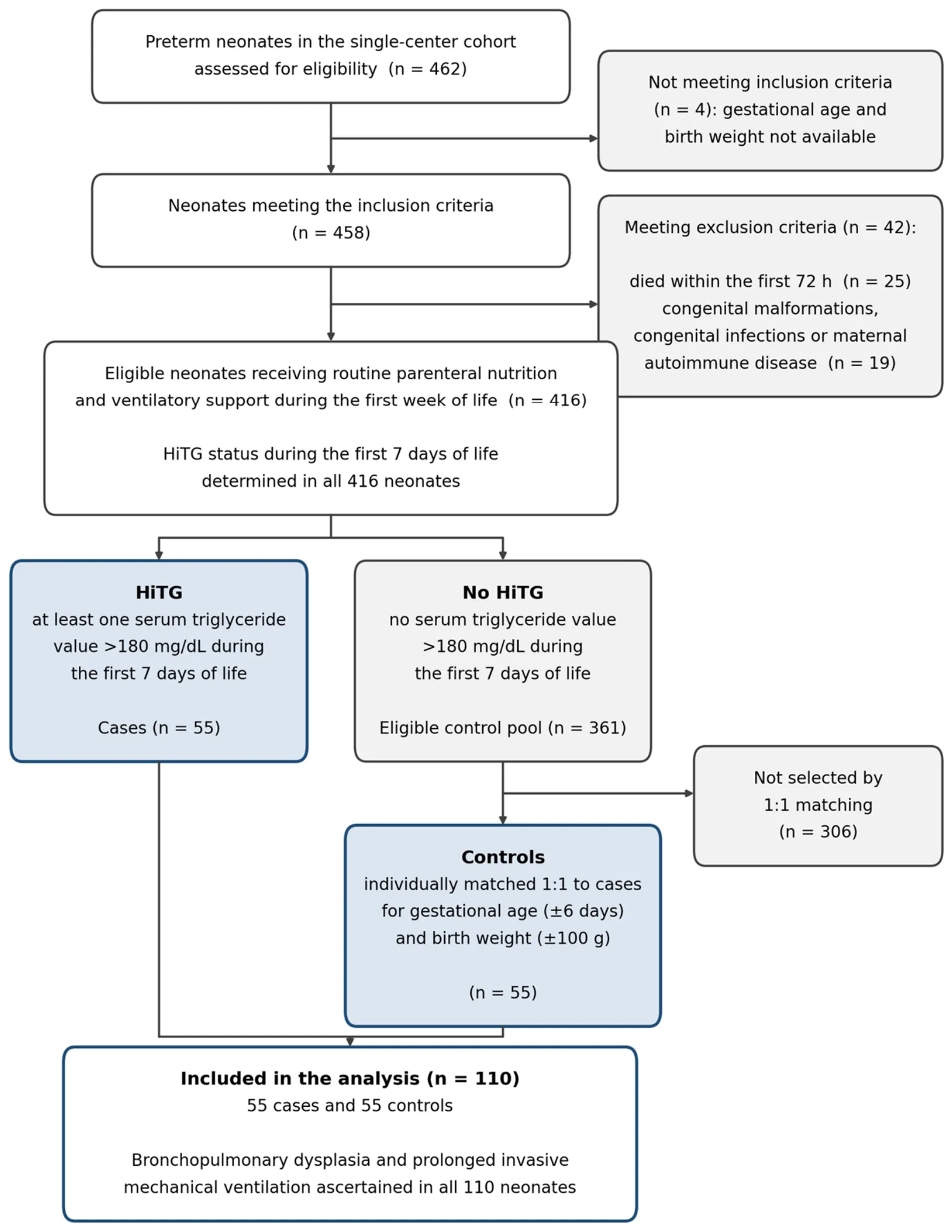
Flow of the study population. Hypertriglyceridemia (HiTG) status during the first 7 days of life was determined in all eligible neonates, in accordance with the unit protocol of routine triglyceride monitoring from the third day of parenteral nutrition. All neonates who developed HiTG during the exposure window were included as cases; controls were individually matched 1:1 to cases for gestational age and birth weight and were drawn from the eligible neonates of the same cohort who did not develop HiTG. Bronchopulmonary dysplasia and prolonged invasive mechanical ventilation were ascertained in all 110 neonates included in the analysis, with no loss to follow-up. Neonates transferred to another hospital within the first 72 h of life and neonates with incomplete clinical records were also ineligible but could not be enumerated separately.

**Figure 2:**
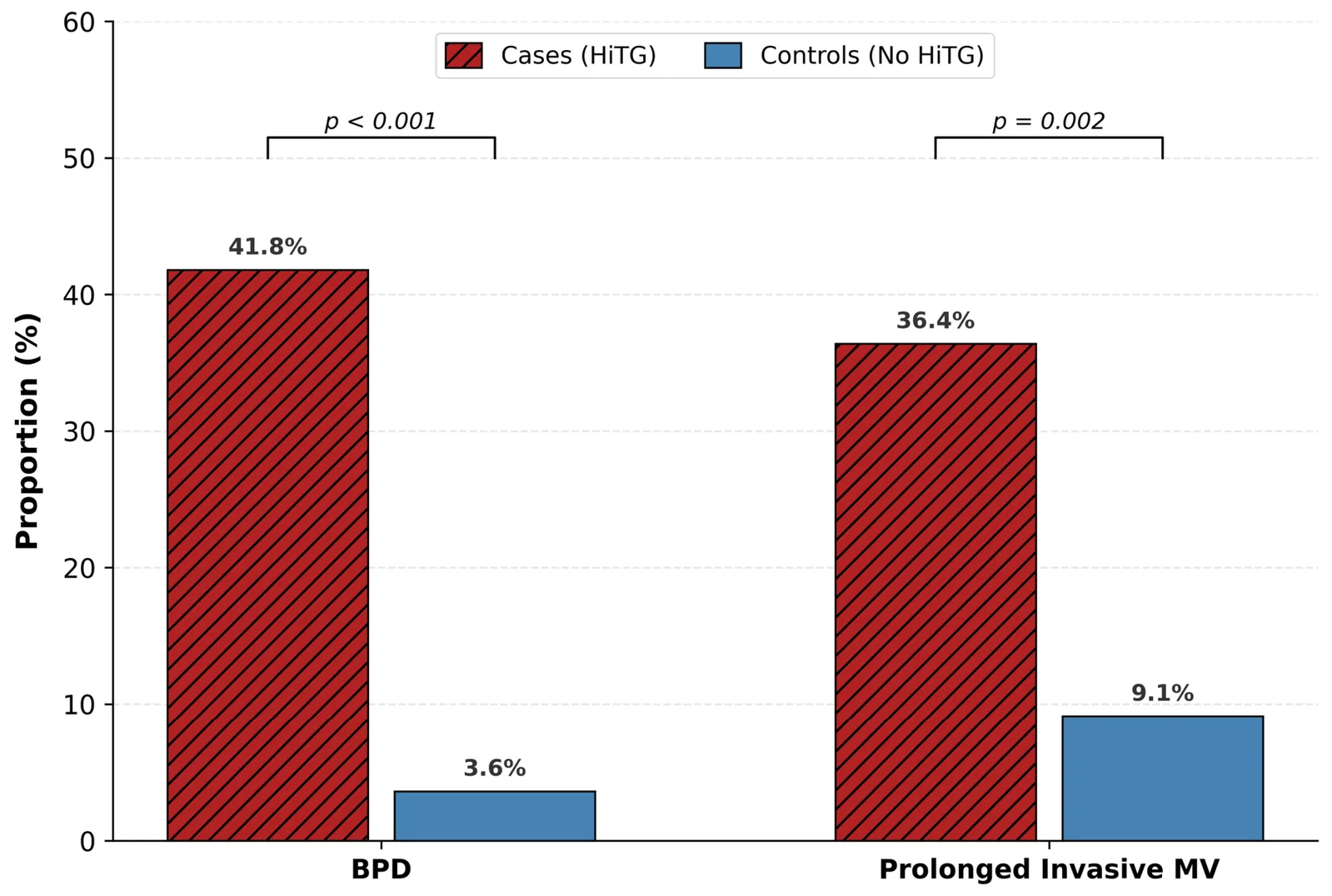
Rate of BPD and pIMV. Bars show the proportion (%) of infants with each outcome. Cases are shown in red; controls in blue. BPD = bronchopulmonary dysplasia; pIMV = prolonged invasive mechanical ventilation.

**Figure 3:**
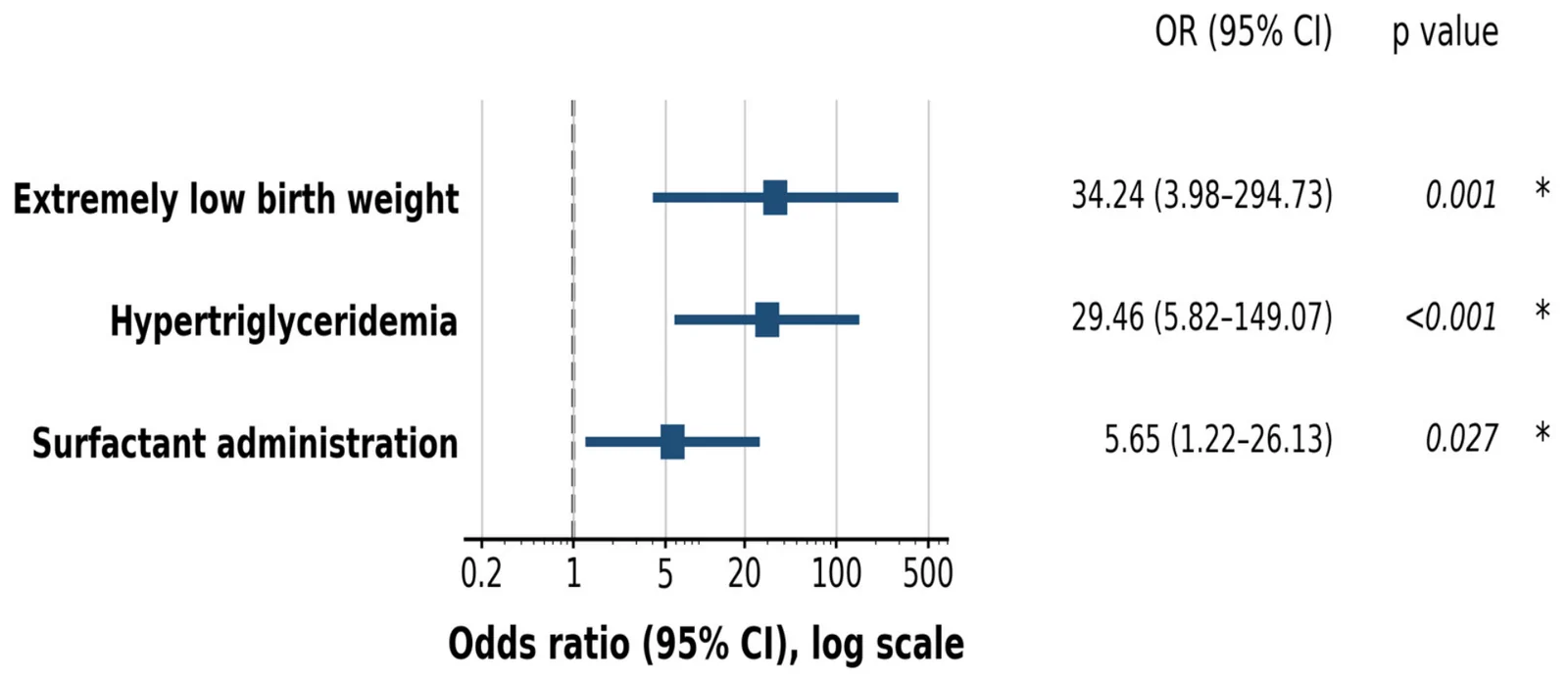
Factors associated with bronchopulmonary dysplasia in preterm infants. Forest plot of adjusted odds ratios (OR) with 95% confidence intervals (95% CI) derived from a multivariable binary logistic regression model with BPD as the dependent variable. Dichotomous covariates entered in the model were: extremely low birth weight (birth weight < 1000 g), hypertriglyceridemia (defined as at least one serum triglyceride value > 180 mg/dL during the first 7 days of life), and surfactant administration. Squares represent point estimates of the OR; horizontal lines indicate 95% CI. The *x*-axis is displayed on a logarithmic scale, and the vertical dashed line at OR = 1 denotes the null effect. Dark blue squares and annotations marked with an asterisk (*) identify covariates with a statistically significant association (*p* < 0.05); light blue squares denote non-significant covariates. Numeric OR (95% CI) and *p*-values are reported to the right of the plot.

**Figure 4:**
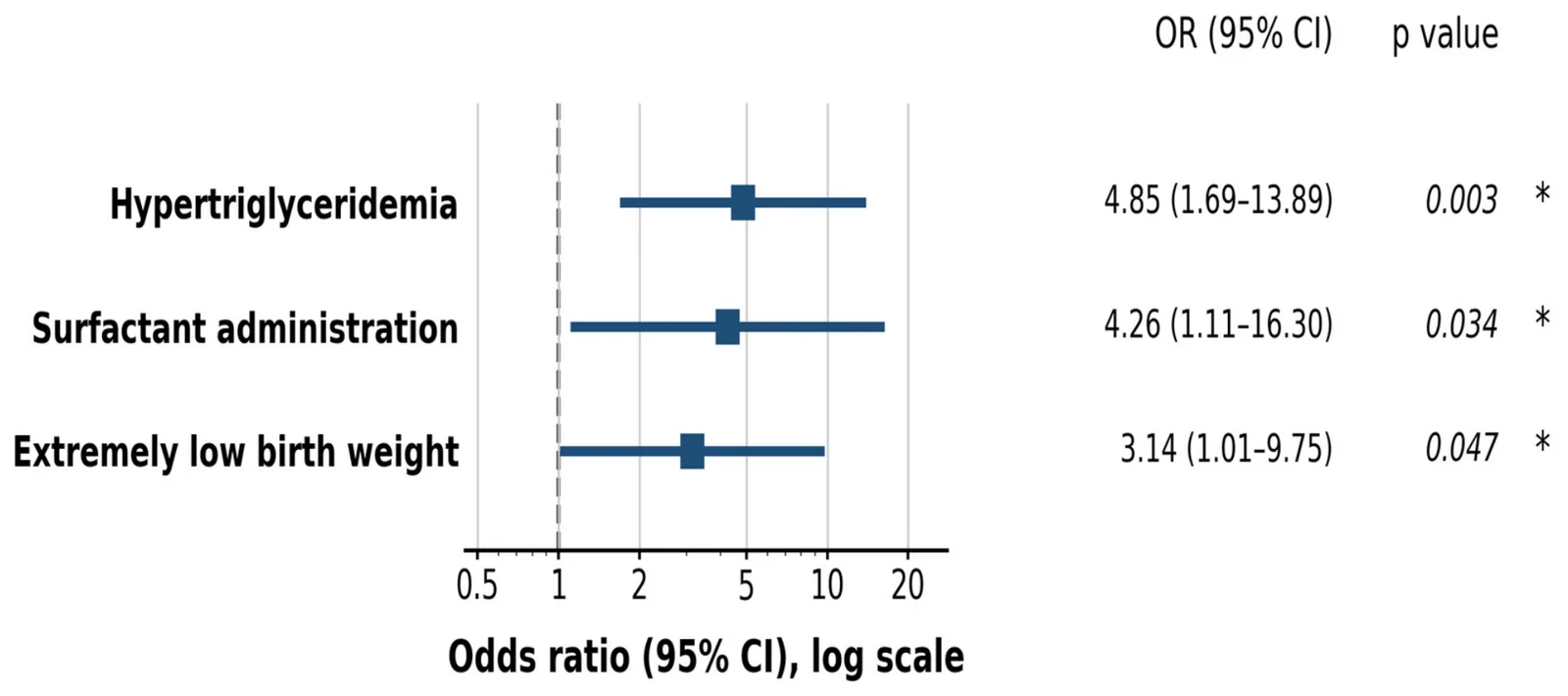
Factors associated with prolonged invasive mechanical ventilation in preterm infants. Forest plot of adjusted odds ratios (OR) with 95% confidence intervals (95% CI) derived from a multivariable binary logistic regression model with pIMV as the dependent variable. Dichotomous covariates entered in the model were: hypertriglyceridemia (defined as at least one serum triglyceride value > 180 mg/dL during the first 7 days of life), surfactant administration, and extremely low birth weight (birth weight < 1000 g). Squares represent point estimates of the OR; horizontal lines indicate 95% CI. The *x*-axis is displayed on a logarithmic scale, and the vertical dashed line at OR = 1 denotes the null effect. Dark blue squares and annotations marked with an asterisk (*) identify the covariates with a statistically significant association (*p* < 0.05); light blue squares denote non-significant covariates. Numeric OR (95% CI) and *p*-values are reported to the right of the plot. Abbreviations: CI, confidence interval; OR, odds ratio.

**Table 1:** Prenatal baseline characteristics of the neonates included in the study.

	Cases	Controls	*p*
	HiTG	NO HiTG	
	*n* = 55	*n* = 55	
Cesarean section, No. (%)	43 (78.2)	48 (87.3)	0.313
Twin pregnancy, No. (%)	14 (25.5)	14 (25.5)	1.000
Maternal age > 35, No. (%)	24 (45.3)	25 (48.1)	0.464
Gestational diabetes, No. (%)	6.0 (10.9)	5 (9.1)	0.500
Pregnancy-induced hypertension, No. (%)	14 (25.5)	18 (32.7)	0.265
Thyroid disorders, No. (%)	7.0 (12.7)	4.0 (7.3)	0.263
Risk of infection ^1^, No. (%)	21 (38.2)	17 (30.9)	0.274
Abnormal doppler velocimetry, No. (%)	15 (27.3)	11 (20.0)	0.251
Placental Abruption, No. (%)	7 (12.7)	6 (10.9)	0.500
Complete antenatal corticosteroids ^2^, No. (%)	29 (52.7)	38 (69.1)	0.059
IUGR ^3^, No. (%)	11 (20.0)	10 (18.2)	0.500

Notes. ^1^ Risk of infection refers to the presence of at least one of the following maternal conditions associated with increased risk of early-onset neonatal sepsis: preterm premature rupture of membranes (pPROM), chorioamnionitis, inadequate intrapartum antibiotic prophylaxis, or maternal Group B Streptococcus (GBS) urinary tract colonization [37]; ^2^ Antenatal corticosteroids refers to a complete course of antenatal steroid prophylaxis [38]; ^3^ IUGR: intrauterine growth restriction.

**Table 2:** Postnatal baseline characteristics of the neonates included in the study.

	Cases	Controls	*p*	Standardized Difference
	HiTG	NO HiTG		
	*n* = 55	*n* = 55		
Gestational age, weeks	26.95 (26.22 to 27.67)	27.62 (27.04 to 28.19)	0.148	−0.278
Birth weight, g	884 (810 to 959)	945 (879 to 1011)	0.225	−0.233
Birth Weight, Z-score	−0.51 (−0.82 to −0.21)	−0.49 (−0.74 to −0.25)	0.909	−0.022
Birth Length, cm	34.2 (32.9 to 35.5)	34.3 (33.3 to 35.3)	0.941	−0.014
Birth Length, Z-score	−0.58 (−1.07 to −0.07)	−0.55 (−0.88 to −0.23)	0.963	−0.009
HC ^1^ at birth, cm	25.12 (24.15 to 26.09)	25.42 (24.60 to 26.23)	0.642	−0.089
SGA ^2^, No. (%)	13 (23.6)	9 (16.4)	0.238	0.183
ELBW ^3^, No. (%)	36 (65.5)	34 (61.8)	0.843	0.076
Male Gender, No. (%)	28 (50.9)	23 (41.8)	0.222	0.183
Acidosis at birth, No. (%)	13 (24.5)	15 (27.3)	0.458	−0.063
Apgar Score below 5, No. (%)	14 (25.5)	13 (23.6)	0.500	0.042
Surfactant, No. (%)	40 (72.7)	38 (69.1)	0.417	0.080
Caffeine, No. (%)	54 (98.2)	55 (100)	1.000	−0.192
Transfusion, No. (%)	38 (69.1)	29 (52.7)	<0.05 *	0.340

Notes. Continuous variables are expressed as mean (95% CI), unless otherwise specified. ^1^ HC: head circumference; ^2^ SGA: small for gestational age; ^3^ ELBW: extremely low birth weight. Standardized differences were computed on the available observations for each variable. * *p*-value < 0.05.

**Table 3:** Nutritional characteristics of the neonates during the first week of life.

	Cases	Controls	*p*
	HiTG	NO HiTG	
	*n* = 55	*n* = 55	
Amount of EN ^1^, mL/kg	51.3 (28.9 to 73.8)	53.5 (34.2 to 72.6)	0.885
MEF ^2^ 0–7 days, No. (%)	5.0 (9.1)	3.0 (5.5)	0.716
PN ^3^ 0–7 days, mL/kg/week	698.4 (659.0 to 737.7)	736.7 (692.4 to 781.1)	0.198
PN ^4^ Carbohydrates, g/kg/week	69.8 (61.4 to 78.2)	73.0 (61.6 to 84.4)	0.652
PN ^5^ Amino acids, g/kg/week	22.3 (19.4 to 25.2)	22.9 (19.5 to 26.2)	0.780
PN ^6^ Lipids, g/kg/week	16.7 (14.7 to 18.7)	19.1 (16.4 to 21.7)	0.152

Notes. Continuous variables are expressed as mean (95% CI), unless otherwise specified. Volumes of parenteral and enteral nutrition are expressed as ml/kg, whereas macronutrient intakes are expressed as g/kg over the first week of life. ^1^ Amount of EN: cumulative amount of enteral nutrition (EN) received during the first week of life; ^2^ MEF: minimal enteral feeding > 175 mL/kg in the first week; ^3^ PN: amount of parenteral nutrition in the first week of life; ^4^ PN Carbohydrates: amount of Carbohydrates administered via PN during the first week of life; ^5^ PN Amino acids: amount of Amino acids administered via PN during the first week of life; ^6^ PN Lipids: amount of Lipids administered via PN during the first week of life.

**Table 4:** Major morbidities of the neonates included in the study.

	Cases	Controls	*p*-Value
	HiTG	NO HiTG	
	*n* = 55	*n* = 55	
Morbidity ^1^, No. (%)	49 (89.1)	41 (74.5)	0.041 *
Exitus, No. (%)	5(9.1)	6 (10.9)	0.500
NEC ^2^ ≥ stage II, No. (%)	2 (3.6)	4 (7.3)	0.679
Late-onset sepsis (clinical and/or culture-proven) ^3^, No. (%)	21 (38.2)	14 (25.5)	0.110
Early-onset culture-proven sepsis, No. (%)	3 (5.5)	3 (5.5)	1.000
Late-onset culture-proven sepsis, No. (%)	13 (23.6)	10 (18.2)	0.640
ROP ^4^, No. (%)	17 (30.9)	26 (47.3)	0.05
PVL ^5^, No. (%)	2.0 (3.6)	1.0 (1.8)	1.000
IVH ^6^ ≥ stage III, No. (%)	7.0 (12.7)	3 (5.5)	0.320
PDA ^7^, No. (%)	34 (61.8)	25 (45.5)	0.063
EUGR ^8^, No. (%)	36 (65.5)	32 (58.2)	0.278

Notes. Continuous variables are expressed as mean (95% CI), unless otherwise specified. ^1^ Morbidity: at least one of the following morbidities: NEC, BPD, ROP, PVL, IVH, sepsis; ^2^ NEC: necrotizing enterocolitis; ^3^ Sepsis: late-onset sepsis, defined as clinical sepsis and/or positive blood culture occurring after 72 h of life; ^4^ ROP: Retinopathy of Prematurity; ^5^ PVL: Periventricular Leukomalacia; ^6^ IVH: Intraventricular Hemorrhage; ^7^ PDA: hemodynamically significant Patent Ductus Arteriosus; ^8^ EUGR: Extrauterine Growth Restriction. Early-onset and late-onset sepsis were distinguished by onset before or after 72 h of life; the age at onset was not available for 3 of the 32 infants with culture-proven sepsis, who could not be classified. * *p* < 0.05.

## Data Availability

The datasets analyzed during the current study are available from the corresponding author upon reasonable request, due to privacy.

## Supplementary Materials

The following supporting information can be downloaded at: https://www.mdpi.com/article/10.3390/nu18193163/s1, Table S1: Published studies on hypertriglyceridemia, intravenous lipid dose and respiratory outcome in very preterm or very low birth weight infants.
