# Supplementary material for: Early Hypertriglyceridemia and Respiratory Outcomes in Preterm Neonates
Source: Nutrients. 2026 Sep 25;18(19):3163. doi: 10.3390/nu18193163 (PMC13667216; doi:10.3390/nu18193163)
Supplement: Supplementary file 1 [file nutrients-18-03163-s001.zip › nutrients-4524653-supplementary.pdf]

Table S1. Published studies on hypertriglyceridemia, intravenous lipid dose and respiratory outcome in very preterm or very low birth weight infants. Studies are ordered by the maximum intravenous lipid dose administered.

Study	Design	Population	n	Definition of HiTG	Intravenous lipid dose (initial → maximum)	Respiratory outcome assessed	Association with respiratory outcome
Sosenko et al., 1993 [15]	Randomized controlled trial (early vs. delayed lipid)	Birth weight 600–1000 g, ventilator-dependent	133 (70 lipid / 63 control)	Not applicable (dose-allocation trial)	0.5 → 1.5 g/kg/day, from <12 h of life through day 7	Chronic lung disease (oxygen ≥28 days), oxygen ≥60 days, ventilation requirement	No. No difference in chronic lung disease. More pulmonary haemorrhage and higher mortality in the 600–800 g stratum
Chan et al., 2021 [19]	Multicentre retrospective cohort	All neonates receiving intravenous fat emulsion; the group with TG 180–400 mg/dL had lower gestational age and birth weight	356 (240 / 91 / 25)	Peak TG <180, 180–400 and >400 mg/dL	1–2 → 3.5 g/kg/day (increments of 1 g/kg/day)	Invasive mechanical ventilation, mortality	Yes for TG ≥180 mg/dL vs. <180 mg/dL. No dose–response relationship across lipid dose strata
Boscarino et al., 2021 [1]	Case–control	Gestational age <32 weeks and/or birth weight <1500 g	145 (40 / 105)	TG >150 mg/dL	1 → 3.5 g/kg/day (increments of 0.5–1 g/kg/day) by day 7	Prolonged invasive mechanical ventilation, BPD	Yes for prolonged invasive mechanical ventilation. BPD more frequent in cases (30.0% vs. 14.3%) but not confirmed at multivariable analysis
Suh et al., 2025 [6]	Single-centre retrospective cohort	Gestational age <32 weeks	276 (104 / 172)	TG >200 mg/dL	0.5–1 → 3–3.5 g/kg/day by the end of the first week (up to 4 g/kg/day if additional energy required)	BPD	Yes. BPD in 83.7% of infants with HiTG vs. 34.3% of controls ($p < 0.001$)
Present study	Case–control	Gestational age ≤32 weeks and/or birth weight ≤1500 g, on parenteral nutrition and ventilatory support	110 (55 / 55)	At least one TG value >180 mg/dL during the first 7 days of life	1 → 3.5 g/kg/day (increments of 0.5–1 g/kg/day) by day 7	BPD, prolonged invasive mechanical ventilation	Yes for both outcomes at multivariable analysis
Pereira et al., 1980 [4]	Comparative study (infants <1 week vs. 2–3 weeks of age)	Appropriate-for-gestational-age preterm infants, birth weight 0.77–1.89 kg	18	Peak plasma TG; no threshold applied	1 g/kg infused over 4 h, i.e. 0.25 g/kg/h (equivalent to 6 g/kg/day if sustained)	Arterial oxygen tension and pulmonary mechanics during lipid infusion	Yes for a significant fall in PaO_2 in infants <1 week of age. BPD and duration of mechanical ventilation were not assessed

Notes. BPD, bronchopulmonary dysplasia; HiTG, hypertriglyceridemia; TG, triglycerides. Doses are those reported in the respective publications. For Pereira et al. the dose is reported as administered, that is 1 g/kg over a four-hour infusion; the corresponding hourly rate and its 24-hour equivalent are given in parentheses to allow comparison with the studies that report a daily dose. Numbers in square brackets refer to the reference list of the main manuscript.
